# A Markov model of urban evolution: Neighbourhood change as a complex process

**DOI:** 10.1371/journal.pone.0245357

**Published:** 2021-01-15

**Authors:** Daniel Silver, Thiago H. Silva

**Affiliations:** 1 Sociology, University of Toronto, Toronto, Canada; 2 School of Cities, University of Toronto, Toronto, Canada; 3 Informatics, Universidade Tecnologica Federal do Parana, Curitiba, Brazil; Peking University Shenzhen Graduate School, CHINA

## Abstract

This paper seeks to advance neighbourhood change research and complexity theories of cities by developing and exploring a Markov model of socio-spatial neighbourhood evolution in Toronto, Canada. First, we classify Toronto neighbourhoods into distinct groups using established geodemographic segmentation techniques, a relatively novel application in this geographic setting. Extending previous studies, we pursue a hierarchical approach to classifying neighbourhoods that situates many neighbourhood types within the city’s broader structure. Our hierarchical approach is able to incorporate a richer set of types than most past research and allows us to study how neighbourhoods’ positions within this hierarchy shape their trajectories of change. Second, we use Markov models to identify generative processes that produce patterns of change in the city’s distribution of neighbourhood types. Moreover, we add a spatial component to the Markov process to uncover the extent to which change in one type of neighbourhood depends on the character of nearby neighbourhoods. In contrast to the few studies that have explored Markov models in this research tradition, we validate the model’s predictive power. Third, we demonstrate how to use such models in theoretical scenarios considering the impact on the city’s predicted evolutionary trajectory when existing probabilities of neighbourhood transitions or distributions of neighbourhood types would hypothetically change. Markov models of transition patterns prove to be highly accurate in predicting the final distribution of neighbourhood types. Counterfactual scenarios empirically demonstrate urban complexity: small initial changes reverberate throughout the system, and unfold differently depending on their initial geographic distribution. These scenarios show the value of complexity as a framework for interpreting data and guiding scenario-based planning exercises.

## Introduction

This study endeavours to build and explore a Markov model of socio-spatial neighbourhood evolution, taking into account that cities are complex systems. Using Toronto, Canada, as our case study, we join and extend insights from two related literatures that have largely developed in parallel: neighbourhood change research and complexity theories of cities. Building on these traditions, we pursue three research questions: 1) What are the main types of neighbourhoods in the city, and how are they organized, both spatially and hierarchically? 2) What are the main patterns of neighbourhood change over time, and how are they inflected by space? 3) How are these trends likely to unfold in the future, and how might they change under certain urban planning scenarios?

To answer these questions, first, we examine publicly available quinquennial census data from 1996-2016 to classify Toronto neighbourhoods into distinct groups using established geodemographic segmentation techniques, a city where this is a relatively novel application. As the fastest growing city in North America [1] and among the most ethnically diverse cities in the world [2], Toronto offers an important research site. While Toronto neighbourhoods have been widely studied [3], the more holistic approach afforded by these methods has been largely missing, as most studies rely on changes in single variables or indexes [4, 5]. Where neighbourhood typologies have been explored [3], this has typically been restricted to a small number of types without examining changes among those types but instead looking at how fixed types change over time with respect to specific indicators like income or ethnic composition. To mitigate the difficulty of qualitatively analyzing a higher number of neighbourhood types, we situate many neighbourhood types within a hierarchical classification. While multilevel regression models and clustering models are common in the field (e.g., [6, 7]), our approach differs in that we examine a hierarchy of neighbourhood types that situates (13) more specific types within (3) more general neighbourhood categories. This approach enables us to show, for instance, that significant temporal patterns of change reveal a unique spatial structure where changes are focused at interstitial zones in the classification scheme.

Second, we use Markov models to identify generative processes that produce patterns of change in the city’s distribution of neighbourhood types. We add a spatial component to the Markov process to uncover the extent to which change in one type of neighbourhood depends on nearby neighbourhoods’ character. In contrast to prior studies in this research tradition that have explored Markov models, we validate the model’s predictive power. More specifically, we predict the future distribution of neighbourhood types based on their past distribution and transition probabilities. Markov models of transition patterns prove to be highly accurate, successfully predicting the final distribution of neighbourhood types with low error—an average RMSE of 0.008. Examining these models shows that the predominant temporal pattern is continuity, as most neighbourhood types tend to reproduce themselves over time. However, this tendency is reduced in the city’s interstitial areas. Spatial Markov models help to understand more precisely the extent to which neighbourhood evolution is influenced by its surrounding areas. In our context, they show a deep interconnection between urban space and time.

Finally, we demonstrate how to use such models in theoretical scenarios considering the impact on the city’s predicted evolutionary trajectory when existing probabilities of neighbourhood transitions or distributions of neighbourhood types would hypothetically change. Counterfactual scenarios indicate urban complexity: small initial changes in transition probabilities can lead to broader transformations. We find that if the city were to continue to evolve according to its existing trajectory, the dominant trend would be increased socioeconomic polarization: for example, elite suburban neighbourhoods would increase their overall share. The hypothetical scenarios we explore suggest that small but strategically located changes could theoretically mitigate or reverse this trend. Moreover, when we add spatial dependencies to the model, we find an indication, among others, that the spatial distribution of changes in initial conditions generates divergent evolutionary outcomes.

Our studied scenarios demonstrate in an empirical setting the utility of core concepts discussed in complexity theories of city. If much past work in this tradition has been in a mathematical and simulation context, we bring complexity ideas into dialogue with empirical neighbourhood research and show its value as a heuristic for interpreting data and guiding scenario-based planning. Specifically, we suggest the value of paying attention to threshold effects, non-linearity, and recursivity, that is, the tendency of existing conditions to reproduce themselves and for small changes to have potentially larger effects at critical thresholds. To do so, we build on and extend prior neighbourhood research that has studied non-linear neighbourhood dynamics, using techniques and approaches (such as geodemographic segmentation, Markov models, and counterfactual scenarios) that this research has not yet explored.

Beyond the specific findings, our study seeks to advance research in both socio-spatial neighbourhood change and urban complexity. Our contributions can be summarized as:

- Application of multivariate clustering methods to identify neighbourhood types and their changes in Toronto. In this application, we incorporate the hierarchical (nested) structure of the resulting neighbourhood types into the analysis, which enables us to better understand neighbourhood change among the more specific types in terms of their categorical and spatial position within the hierarchy;
- Development and use of Markov models to identify the generative processes that produce patterns of change, exploiting the formal properties of Markov chains to do so. In addition, we add a spatial component to the Markov process to investigate spatial dependence in different contexts. We validate the predictive power of the model and, having done so, demonstrate how to use such models to evaluate hypothetical scenarios to improve knowledge about how the city would evolve under different conditions;
- Regarding complexity theories of city, our experiments bring complexity ideas into dialogue with empirical neighbourhood research and show its value for interpreting outcomes and guiding scenario-based planning.

We proceed in 4 sections. First, we review related work on neighbourhood change and urban complexity, while also providing relevant background about Toronto. Second, we discuss data and methodology. Third, we report results, organized around the three research questions noted above. We conclude by discussing our results, noting limitations, and considering future directions.

## Related work

This study joins and builds upon two research streams: socio-spatial neighbourhood change and complexity theories of cities. In this section, we review key concepts and trends in both, note how our study advances them, and articulate our research questions that emerge from them. We also provide a brief overview of relevant work about our research setting, Toronto, Canada.

### Socio-spatial neighbourhood change research

The tradition of socio-spatial neighbourhood change research extends back roughly a century, to the Chicago and Atlanta schools of urban sociology [8–10]. These early efforts pioneered methods of close observation and data collection, including contributing to the modernization of census bureaus and the creation of standardized census tract boundaries to facilitate neighbourhood comparison [11]. Even if they have been criticized and modified over time, many of the key concepts and typologies from this period remain relevant and in circulation today [12]. These include the concentric zone model, in which groups compete for prime central space and change radiates out from there [13], or the neighbourhood succession and filtering model in which neighbourhoods defined by upper status groups are replaced in cycles by lower status groups [14–16].

Mid-century researchers carried this tradition forward with the aid of novel statistical techniques. Shevsky and Bell [17], in particular, elaborated factorial ecology as a research paradigm for multivariate classification of neighbourhood types and change [17]. Factorial ecology utilized factor analysis to reduce many variables to a small number of components, which in turn were used to characterize neighbourhoods and their changes (see [18, 19]). This research suggests that neighbourhoods could be differentiated along three primary dimensions, which summarize their economic, family, and ethnic status. While contemporary research often adds additional factors such as housing, transit, and built form [16], a key legacy of factorial ecology is to incorporate variables describing these dimensions [20–24].

If the multidimensional character of factorial ecology never entirely disappeared from academic research, it fell by the wayside for several decades. Instead, neighbourhood and urban change research tended to focus on single variables, often in the form of segregation indices [25]. Nevertheless, largely in market research, geodemographics emerged in this period and promoted cluster analysis as an alternative approach to detecting neighbourhood types and change as complex combinations of many variables [26, 27]. In particular, geodemographic researchers argued that cluster analysis could detect both major types and important but rare types that tend to be lost in factor analysis.

In recent decades, these geodemographic techniques began to appear more in an academic context [28–30]. A key advance was to perform the cluster analysis on many variables across multiple time points, and then to compare transitions over time across the resulting neighbourhood types [7, 31–33]. While these studies failed to exploit the analytical potentials in the underlying transition matrices, they did generate some important results, primarily with regard to US neighbourhoods: Stability (including persistent poverty) is much more common than change. Polarization over time (declining middle class neighbourhoods) is a general tendency. Neighbourhood succession (downgrading) does not necessarily follow the linear trajectory envisioned by earlier theorists, and varies greatly by city. Upgrading (including gentrification) occurs less frequently than one might imagine from popular discussions and is focalized in ethnically diverse neighbourhoods located in a few large metro areas. Finally, this research emphasized the existence of a great diversity of neighbourhood change types, many of which do not neatly fit into the categories of stability, succession, or upgrading in that they involve movement among neighbourhood types with similar socioeconomic status.

More recent studies of socio-spatial neighbourhood change have advanced in formal sophistication as well as geographic and temporal scope. The work of Delmelle (e.g., [34]) has moved the field forward decisively in these regards. Perhaps most importantly, this has occurred by making neighbourhoods’ longer-term temporal trajectories the primary unit of analysis and adapting sequence analysis techniques from genomics (see also [35]). This work largely confirmed key substantive results from previous US studies regarding the main patterns of change. However, it added by locating temporal pathways in space, finding, for instance, that high poverty minority and wealthy white neighbourhoods expand in a relatively contiguous pattern. In contrast, multiethnic areas grow in a more dispersed pattern.

Current research tends to focus on the nature, patterns, and causes of change itself [36]. Most use some form of cluster analysis, but there are several ongoing debates [37]. Authors often question the merits of prototype-based approaches (i.e. k-means) or hierarchical approaches [25]. There are also discussions about the value of sequence analysis or Markovian approaches. Advocates of sequence analysis [34] note that first-order Markov chains disconnect singular transitions from the broader pattern of which they are a part and cannot distinguish later vs. earlier transitions of the same type (e.g., ABAAAA looks the same as AAAABA). Advocates of Markovian approaches argue that sequence analysis misses the generative processes that produce trajectories and that the results of sequence analysis are highly unstable, as they are very sensitive to the particular algorithms used [38]. Also, Markov chains allow predictions to be made regarding future outcomes based solely on its present state, and such predictions are often just as good as the ones considering the full history of the process [39]. These predictions moreover can be made under counterfactual scenarios, either by changing the transition probabilities or the initial distribution of states, thus enabling the researcher to study possible outcomes of new policies, change of preferences, and the like. One drawback of using Markov chains for this procedure is that they do not allow for the emergence of possible new neighbourhood types.

Finally, a common thread in current research involves efforts to spatialize time and temporalize space, either by mapping temporal processes or by pursuing more formal techniques such as spatial Markov analysis [40] for examining how temporal change shifts across geographic contexts. In fact, geographic contexts have proven to be of significant importance in helping to understand neighbourhood dynamics [41, 42]. In particular, Delmelle et al. [41] investigated whether a neighbourhood’s geographic situation impacts its probability of improvement and decline concerning its quality of life profile. To tackle this dependence question, the authors explore a spatial Markov model [40]. Although traditional Markov models have been explored in different contexts, including urban dynamics [42], they miss capturing spatial influences on the process under study. Delmelle et al. [41] observed that declining or improving quality of life is not spatially independent, i.e., surrounding neighbourhoods have clear impacts on a neighbourhood’s probability of improved or diminished quality of life.

Our study continues and extends these past related works. Like most recent work, we use cluster analysis to classify neighbourhood types and examine transitions through those types. We differ by incorporating the hierarchical (nested) structure of the resulting neighbourhood typology into the analysis. This approach enables us to meaningfully include more clusters than is typical in the literature. Similarly, we carry forward the tradition of studying transition probabilities and examining spatial patterns of change, but add by mapping transition rates themselves (rather than sequences). Likewise, similar to the work noted above, we examine the impact of spatial context through spatial Markov chains. Perhaps most significantly, however, in contrast to past work, we exploit Markov models’ formal properties to evaluate their predictive power based on real data and consider the effects of various counterfactual scenarios. This innovation is carried out in both the spatial and non-spatial cases. Here we highlight concepts such as thresholds and non-linearity that are characteristic of complexity theories of cities.

### Cities as complex systems

While socio-spatial neighbourhood change research has often been very empirically focused, primarily through its historical legacy to the Chicago School of urban sociology, it has maintained a theoretical basis that points toward key concepts from complexity thinking. Abbott [43] has stressed this theoretical linkage, noting the importance in this tradition of “turning points” (similar to non-linearity and tipping points), fractal patterns, the emergence of global order from local interaction, and the deep integration of space and time [43].

Many of the insights driving efforts to conceive cities as complex systems derive from the work of Jane Jacobs and Christopher Alexander in the 1960s. The core ideas researchers took from these authors included the notion that behind the seeming disorder and diversity of urban forms, there are strong patterns. These emerge from the myriad local decisions and behaviours by individual actors and organizations [44]. These processes of self-organization suggested treating cities as complex systems. They exhibit emergent orders that require significant energy to sustain, patterns of segregation rooted in competition over space, and punctuated equilibria [45]. In particular, complexity studies of cities have tended to emphasize how self-organization involves three major steps [46]: emergence (where local interactions between parts give rise to complex global structures); steady states (where the system maintains itself recursively); and bifurcation or phase transition (in which the old equilibrium dissolves and a new one emerges).

The major contribution of complexity theories of cities has been primarily theoretical. Researchers have sought to bring diverse urban phenomena, often considered independently, into a common framework [46]. Much work, in particular, has sought to elaborate the mathematical basis for this synthesis. These have joined with considerable use of computer simulation models, especially Agent Based Models and Cellular Automata [44, 47], to demonstrate self-organization and emergence in general and highlight some of their specific urban patterns (such as larger zones of stability and local persistent areas of experimentation and change at their boundaries [48]. At the same time, the sorts of long-term empirical studies of local and multilevel change characteristic of socio-spatial neighbourhood change research are relatively rare (though see [49]).

While much of this work unfolded in a mode of rapid theoretical and methodological growth, more recent studies start from the point of view that complexity theories of cities have “come of age” [50]. They are a relatively mature set of theories and methods, and the main challenges involve clarification, application, and refinement. An area of particular concern involves demonstrating the relevance of complexity ideas to substantive areas of urban studies to avoid the fate of past quantitative research paradigms, which in pursuing narrow technical questions lost sight of larger concerns [51]. Heavy reliance on simulations geared toward short-term predictions similarly makes considering longer-term transformations difficult, leading some researchers to develop more explicitly evolutionary models [52].

A related concern involves challenges in importing assumptions from physics into urban studies, given that cities are composed of human agents that are themselves highly complex cognitive-emotional entities that react to changes in their environments [53]. This has made incorporating complexity theories into urban planning programs difficult, in that complexity stresses a certain degree of unpredictability, non-linearity, and multiple forking paths that are not easily assimilated into clear planning interventions on the model of an engineer pulling levers in a machine [54]. In general, complexity theories caution humility in planning; they also seek to rethink the nature of planning theory to some degree. In particular, some advocate scenario based thinking that considers possible futures under different assumptions, while incorporating sensitivity to the notion that small initial differences can compound into large changes over time [50, 55].

Neighbourhood change research has for its part investigated phenomena characteristic of urban complexity, in particular threshold effects and non-linearity. Galster [56, 57] summarizes much of this work, highlighting key theorized models of the underlying behavioural processes (such as socialization, gaming, tolerance, contagion, and diminishing returns) as well as empirical evidence of non-linear neighbourhood dynamics. This empirical work typically uses regression-based techniques on single variables or indexes to examine whether there are critical thresholds where changes in variables such as poverty rates generate larger changes at later time periods. Overall, past research has found the most striking thresholds at extremes, such as at the top 10% of poverty rates and has suggested policy value in targeted “remedial” interventions for neighbourhoods approaching potentially dangerous tipping points [56].

Our study draws from complexity theories of cities primarily as a heuristic guide toward interpreting the results of our analyses and formulating our questions. More specifically, considering cities as complex systems leads us to treat neighbourhood change as a multilevel process in which transitions are constrained by a vertical hierarchy but also occur horizontally across higher-order boundaries. Similarly, we seek to locate persistent patterns of stability and volatility. Perhaps most importantly, we adopt the scenario-based approach as a way to imagine possible urban futures. Here we formulate our scenarios in the light of complexity theory by considering hypothetical changes in the transition probabilities of neighbourhood types strategically located at the boundaries among its higher order structuring principles. Crucial as well is our emphasis on how small changes can have large consequences that reverberate throughout the system, affecting other parts indirectly. In contrast to prior work reviewed by Galster [56, 57] that primarily used regression-based models, single variables or indexes, and sought to identify remedial forms of intervention, we use Markov models and multivariate classification techniques to envision counterfactual scenarios and identify where small introductions of rare transitions might mitigate or reverse larger city-wide trajectories toward growing polarization.

### Contextualizing the case of Toronto

Our study uses Toronto, Canada, as a case study for examining socio-spatial neighbourhood change within a complex urban setting. Toronto has received significant attention from urban researchers in recent years, in large part because of its rapid growth and dramatic demographic transformations. In 2019, Toronto was the fastest growing city in North America, gaining nearly as many new residents as New York lost [1] and especially since the 1980s international migration from East and South Asia, Africa, the Caribbean, and the middle East have made it one of the world’s most ethnically diverse multicultural cities [2].

Amidst these broader metropolitan changes, scholars have turned their attention to neighbourhood change. The vast majority of this research does not use multivariate geodemographic classification techniques but rather examines neighbourhood trends in single variables or indexes, such as income, occupation, or ethnicity, e.g., [4, 5]. This research has identified a long-term economic decline of the suburban zone relative to a gentrifying core, marked by the growth of young professional and “creative class” enclaves in the latter [58, 59], and growth in lower income non-European immigrant groups in the former [60], often concentrated in its ageing social housing high-rise towers and alongside or nearby older Southern and Eastern European communities. These are generally located in the city’s Northeast and Northwest areas where rapid transit service is relatively poor, whereas older established upper status suburban neighbourhoods generally follow the subway lines.

Still, some research has used multivariate classification approaches to identify neighbourhood types in Toronto, such as Principal Component Analysis [3], Self Organizing Maps [61], or cluster analysis [35]. This and similar univariate work [58] tends to find that a three-part structure has largely persisted even amidst the city’s ongoing transformations: a dense downtown core largely populated by young people and highly educated “creative class” professionals and aspirants; traditional suburban areas with large single family homes often inhabited by members of Canada’s historical Protestant elite, Toronto’s main Jewish areas, as well as areas settled by newer Chinese immigrants; a relatively marginalized inner suburban areas in the Northeast and Northwest populated primarily by lower income non-white residents (in particular South Asian, Black, and Arab) along with older white, often Italian, working class communities. In this core-periphery pattern, Toronto resembles other large post-industrial cities such as London, UK [3].

Our study builds on and extends this multivariate research. By taking a multilevel, hierarchical approach, we incorporate the three-part structure identified by past research as a more general structuring principle organizing the city’s pattern of neighbourhood types. But by nesting more specific types within this structure, we are able to incorporate a richer set of types than past research. Moreover, again in contrast to the few studies that have examined change across multivariate neighbourhood types (e.g. [35]), this nested approach allows us to study how neighbourhoods’ positions within this structure shape their trajectories of change. Finally, no prior studies of multivariate neighbourhood types of which we are aware have used Markov chains to predict neighbourhood trajectories in Toronto, study the spatial dependency of change across neighbourhood types, or as a basis for conducting counterfactual thought experiments to explore possible futures as informed by ideas from complexity theories of cites. By filling this gap, we add an important case to the broader literature on socio-spatial neighbourhood change.

Synthesizing and extending the work reviewed above, this study pursues three interrelated research questions. We (i) extend existing work on geodemographic segmentation to Toronto and identify its main types of neighbourhoods and their organization (spatially and hierarchically); (ii) extend prior use of Markov models by validating their accuracy and using them to examine key patterns of neighbourhood change over time, and their spatial dependency; and (iii) pursue ideas from complexity theory by developing counterfactual experiments to determine how these trends would change under different hypothetical scenarios, paying attention to non-linear dynamics.

## Data and methodology

### Data sources and preprocessing

We use publicly available data from 1996-2016 for census tracts located within the Toronto Census District (CD) [62] representing five different points in time *t* ∈ *T* = {1, 2, 3, 4, 5}. Consistent with past studies [7, 16, 31–33], we classify neighbourhoods according to a broad set of demographic, socioeconomic, and housing characteristics (all variables used is in Table 1).

**Table 1:** Variables used in the analysis.

Variable Type	Variable Label	Variable Description
**Socioeconomic**	marmarried	% married
	unemp	% unemployed
	labmanag	% management occupations
	labartsport	% arts, recereation, and sports occupations
	labbusi	% business, finance, and administrative occupations
	labsci	% natural and applied science occupations
	labserv	% sales and service occupations
	labblue	% blue collor occupations (trades + manufacturing)
	commauto	% drive to work
	incavhh	average household income
	educba	% with BA degree or higher
	incgov	% of income from government transfer
	popdens	population density
**Demographic**	ageyouth	% population 25-34
	agesenior	% population 65 over
	vmblack	% black visible minority
	vm.sasia	% south asian visible minority
	vm.chi	% chinese visible minority
	vm.arab	% arab visible minority
	eth.ital	% italian ethnicity
	eth.port	% portuguese ethnicity
	eth.grk	% greek ethnicity
	eth.jew	% jewish ethnicity
**Housing**	dwdetach	% detached housing
	dwhirise	% apartments over 5 stories
	tenurerenters	% renters
	dwellval	average dwelling value
	move5	% moved in past 5 years

As census-tracts boundaries can change over time, a fundamental challenge when making a longitudinal comparison of spatial data is to ensure that boundaries are consistent. Our dataset minimizes this problem by utilizing the methodology proposed by Allen and Taylor [63]. This approach harmonizes census-tracts to a common set of boundaries, using map-matching, dasymetric overlays, and population-weighted interpolation. Some census-tracts did not contain data for some variables, and for the purposes of this analysis they were removed from the dataset (2.5% of all tracts). In total, we study 514 census-tracts: *c*_*i*_ ∈ *C* = {*c*_1_, *c*_2_, …, *c*_514_}. We represent each census-tract on the n-dimensional space $V^{c,t}=\left\{v_{1}^{c,t},v_{2}^{c,t},\ldots ,v_{n}^{c,t}\right\}$ defined by all the *n* = 28 census variables. Next, all variables are normalized by z-score each year to control for different measurement scales and to enable a fair comparison of changes across time. In other words, we create a new vector $F^{c,t}=\left\{f_{1}^{c,t},f_{2}^{c,t},\ldots ,f_{n}^{c,t}\right\}$, where each variable $f_{i}^{c,t}=\left(v_{i}^{c,t}-\mu^{c,t}\right)/\sigma^{c,t}$, with *μ*^*c*,*t*^ and *σ*^*c*,*t*^ being the average and standard deviation of *V*^*c*,*t*^, respectively. Thus, *F*^*c*,*t*^ represents a specific tract *c* on the census year *t*. In this way, the normalized variables in *F*, therefore, represent a relative value compared to all other values in the city in a particular year.

### Overall workflow

**Fig 1:**
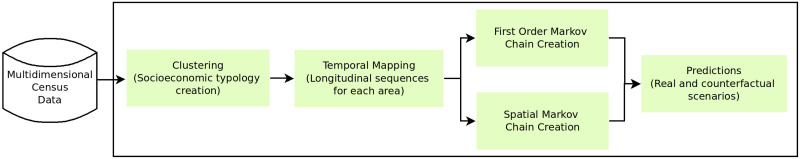
Overall workflow.

Our workflow is in part inspired by the work of Elizabeth Delmelle [34], regarding the way clustering is used to extract socioeconomic typologies, though it is consistent with other work in the field [34]. Fig 1 summarizes our main methodological steps, explained in the next sections.

### Socioeconomic typology and temporal mapping

In order to create a longitudinal socioeconomic typology for the census-tracts under evaluation, we perform a clustering process on all census-tracts *c* ∈ *C* represented by *F*^*c*,*t*^ observed on the five years studied. Most neighbourhood classification studies use either a prototype-based clustering (typically k-means) or hierarchical clustering [25], with ongoing debates about their relative merits [7]. In our study, we strike a middle ground by combining both approaches. We use a k-means clustering algorithm to partition our data. However, instead of randomly choosing the initial centroids, which makes the results sensitive to these choices, we select these centroids based on hierarchical clustering (using Euclidean distance with Ward linkage criteria) [64]. This approach is also known as hierarchical k-means. This hybrid method has the benefit of producing a stable solution in a computationally efficient way, while also allowing us to examine the general organization of the clusters within the resulting hierarchical data space. This, in turn, can also guide decisions about the number of clusters to use in the analysis based on a dendrogram.

Following the clustering procedure, we interpret the results according to procedures typical in the neighbourhood classification literature. We use radar plots to identify each cluster’s semantic based on their centers’ most prominent variables. Each cluster then receives a unique name *u* ∈ *U* that tries to express its latent meaning. While these names are helpful in making sense of the analysis, their subjective nature does not compromise the results—using different names as labels could provide a similar message. Each tract is then assigned one of those labels five times (each one representing a point in time corresponding to a census year). Finally, a sequence for each tract is created, depicting its longitudinal trajectory through the multidimensional neighbourhood typology: *longitudinal trajectory tract*_*i*_ = (*u*_*t*=1_, *u*_*t*=2_, *u*_*t*=3_, *u*_*t*=4_, *u*_*t*=5_), for the five census years under study. We employ hierarchical clustering and also t-Distributed Stochastic Neighbor Embedding (t-SNE), which is a tool for dimensionality reduction that is well suited for the visualization of high-dimensional data, as in our case [65]. Specifically, we use hierarchical clustering to identify similar neighbourhood types based on the clusters’ centroids and place them in a higher-order grouping (in this study, the three most distinct sets of neighbourhood types), confirming this result with t-SNE.

### Markov models

Having generated a longitudinal trajectory for each census-tract through the neighbourhood types, we create two types of Markov chain: a first order Markov chain and a spatial Markov chain. These tools enable us to study the evolution of neighbourhood types over time.

#### First order Markov chain

Markov chains are stochastic processes that can be parameterized by empirically estimating transition probabilities between discrete states [66]. A random variable *X* represents a sequence of states, where a discrete level on *X* is the state of this variable in a certain time. The Markov chain of the first order, explored in this study, is one for which the probability of *X* being in state *j* (neighbourhood type, in our case) at time *t* depends only on the state immediately preceding one *i* of *X*, at time *t* − 1. For *K* states, the first order transition matrix has a size of *K* × *K*, where *K* is the size of the number of unique neighbourhoods types (|*U*|) and takes the form:

$$\begin{array}{c} \left[\begin{array}{cccc} m_{1,1} & m_{1,2} & \cdots & m_{1,K}\\ m_{2,1} & m_{2,2} & \cdots & m_{2,K}\\ \vdots & \vdots & \vdots & \vdots \\ m_{K,1} & m_{K,2} & \cdots & m_{K,K} \end{array}\right] \end{array}$$ (1)

The probabilities from transitioning from state *i* to *j* on *M* can be estimated from the relative frequencies of the transitions on all longitudinal trajectories for all tracts. Thus, each transition probability is:

$$\begin{array}{c} m_{ij}=\sum_{t=1}^{T-1}a_{i^{t},j^{t+1}}/\sum_{t=1}^{T-1}a_{i^{t}}, \end{array}$$ (2)

where $a_{i^{t},j^{t+1}}$ is the amount of neighbourhoods transitioning from type *i* in year *t* to type *j* in *t* + 1, and *a*_*i*^*t*^_ is the total amount of neighbourhoods in type *i* in period *t*.

Before examining this Markov chain, we validate its performance as a predictive model of the city’s evolution. For that, we derive a Markov chain from years 1-4 (1996-2011). Thus, in this Markov chain *t* ∈ *T*′ = {1, 2, 3, 4}, and, consequently, the longitudinal trajectory for each tract is represented by the sequence: (*u*_*t*=1_, *u*_*t*=2_, *u*_*t*=3_, *u*_*t*=4_). We use that new Markov chain to predict the fifth year (2016). For that, we use the probabilities of having each neighbourhood type in one specific year in the past *π*(*t*) = [*π*_1_(*t*), *π*_2_(*t*), …, *π*_*K*_(*t*)], where *i*-th element *π*_*i*_(*t*) represents the probability of a neighbourhood type in year *t* ∈ {1, 2, 3, 4}, to predict *π*(*t* = 5). Thus, we make four distinct predictions for (*t* = 5), each of them considering *π*(*t*) for one of the previous years studied.

We compare the model’s predicted proportions of neighbourhood types to the actual proportions in year five, against two baseline models: a) a *K* × *K* identity matrix, b) 1000 randomly generated first order transition matrices *M*_*x*_, where *x* ∈ {1, 2, …, 1000}. Each *M*_*x*_ is derived from a random order of the set of all longitudinal trajectory tracts, by randomly choosing four neighbourhood types in *U* to create a new sequence for each tract studied: $\left(u_{t=1}^{x},u_{t=2}^{x},u_{t=3}^{x},u_{t=4}^{x}\right)$. We explore *M*_*x*_ to perform predictions about the distribution of neighbourhood types on year 5, *π*(*t* = 5), using for that *π*(*t* = 1), *π*(*t* = 2), *π*(*t* = 3), and *π*(*t* = 4) on each *M*_*x*_. With the identity matrix we disregard the Markov chain, and our prediction of year *π*(*t* = 5) is *π*(*t*), with *t* ∈ {1, 2, 3, 4}.

We also explore M to perform predictions about the distribution of neighbourhood types 50 time steps in the future. To do so, we use *π*(*t* = 5), to make predictions based on *M*. This results in estimations of probabilities of having each neighbourhood type in Toronto in a nearer future than the steady state, which, in our case, requires 646 steps.

#### Spatial Markov chain

Spatial Markov chains allow for a more comprehensive study of the spatial influence of the transitional dynamics [40]. In this study, we investigate whether transition probabilities are dependent on neighbourhood types. Instead of estimating one transition probability matrix, as done in the non-spatial case, spatial Markov chains require estimation of *K* transition probability matrices, each of which is conditional on the neighbourhood type at the year immediately before. A key step in the implementation of a spatial Markov chain is the definition of a neighbourhood’s vicinity. Here we include the five nearest neighbours (a range commonly used in spatial analysis literature [6, 41]), and we calculate conditional probabilities based on those neighbours. A spatial Markov matrix decomposes the non-spatial *K* × *K* transition matrix into a *K* × *K* × *K* system, where *K* is the number of neighbourhood types under study. Thus, we have one transition matrix *K* × *K* for each type of neighbourhood, and each of them represents transition probabilities conditioned on neighbouring a specific neighbourhood type, considering the year immediately before. We represent a spatial Markov for each neighbourhood type *u* ∈ *U* as *S*^*u*^. If a certain row of *S*^*u*^ sums to zero, occurring when a certain neighbourhood type was never a neighbour of the specific one it is being conditioned on, we assign 1 to the position representing the diagonal element to ensure each conditional transition probability matrix, i.e., *S*^*u*^, is a valid stochastic matrix (each row sums up to 1) [40]. In other words, the probability of staying with the same neighbourhood type is 1 in that specific conditional case.

We use *S*^*u*^ to perform predictions about the state of the city 50 steps in the future. To do so, we again use the probabilities of having each neighbourhood type in year five, *π*(*t* = 5), in this case to make predictions based on *S*^*u*^. This results in estimates of the probabilities of having each neighbourhood types in Toronto in the future, *P*^*u*^, conditioned on a certain type of neighbour *u* ∈ *U*.

We also perform a test of spatial independence, comparing the transition probabilities conditioned on neighboring a specific neighbourhood type (spatial case) with transition probabilities considering the entire dataset without this restriction (non-spatial case). For that, the transition probabilities under *H*_0_: ∀_*u*_: *p*_*ij*|*u*_ = *p*_*ij*_(*u* = 1, 2, …, *U*) are contrasted against those under *H*_*a*_: ∃_*u*_: *p*_*ij*|*u*_ ≠ *p*_*ij*_, using a Pearson *χ*^2^ test statistic as presented by Bickenbach and Bode [67]:

$$\begin{array}{c} Q^{\left(M\right)}=\sum_{u=1}^{\left|U\right|}\sum_{i=1}^{\left|U\right|}\sum_{j\in A_{i}}^{}n_{i|u}\frac{\left({\hat{p}}_{ij|u}-\hat{p_{ij}}\right)}{{\hat{p}}_{ij}}∼asy\;\chi^{2}(\sum_{i=1}^{\left|U\right|}\left(a_{i}-1\right)\left(b_{i}-1\right)) \end{array}$$ (3)

where $A_{i|u}=j:{\hat{p}}_{ij|u}>0$ is the set of nonzero transition probabilities in the *i*-th row of the transition matrix estimated from the *u*-th neighbor type, $A_{i}=j:{\hat{p}}_{ij}>0$ is the set of nonzero transition probabilities in the *i*-th row of the transition matrix estimated from the entire dataset (non-spatial), *a*_*i*_ = #*A*_*i*_ is the number of elements in *A*_*i*_, and *b*_*i*_ = #*B*_*i*_ is the number of submatrices (for the spatial case) for which a positive number of observations is available for the *i*-th row [66].

#### Counterfactual scenarios

We also create counterfactuals scenarios to demonstrate the utility of our approach to, for example, guiding urban planning decisions. Thinking in counterfactuals demands envisioning a hypothetical reality that contradicts the known facts, namely census tract data in our case. Here we evaluate the impact of imaginative interventions in neighbourhoods by either changing their transition probabilities or changing their initial conditions.

#### Non-spatial case

Following ideas from complexity theories of cities, we explore the extent to which small initial changes, when repeatedly iterated, can lead to relatively large and sometimes unexpected changes, both direct and indirect. Specifically, we examine different scenarios representing changes λ starting from 1% (λ = 0.01) with 1% increments up to 25% (upper-bound for a valid Markov chain in the interventions considered), to the probability that three neighborhood types—*UP* = {*“black predominant”,“mixed suburban”, “mixed creative”*}—would appear in three entrenched neighborhood types—*DOWN* = {*“elite suburban”, “established creative”, “young urban professional”*}—decreasing their reproduction rates correspondingly by 3 × λ each. Thus, we evaluate 25 scenarios, representing different changes λ_*w*_ = *w* * 0.01, with $\sum_{w=1}^{25}$, in each of them: $\sum_{i=1}^{3}\sum_{j=1}^{3}\left(DOWN_{j}\to UP_{i}\right)+\lambda_{w}$ and $\sum_{i=1}^{3}\left(DOWN_{i}\to DOWN_{i}\right)-\left(3\times \lambda_{w}\right)$, where *X* → *Y*, represents a specific transition on the Markov chain *M*. In this way, we ensure a valid Markov model in this hypothetical scenario. This imagined intervention represents a strategic planning decision to promote interchange among parts of the city that rarely interact and to induce change in some of the city’s most entrenched upper status areas (where reproduction rates are near or above .9). Indeed, transitions between these neighbourhood types are exceedingly rare: all are below 3% and most are near 0. Comparing these scenarios allows us to investigate threshold effects.

#### Spatial case

For this experiment, we imagined a scenario where an intervention in the city changed certain neighbourhoods of the type “towers” to “mixed suburban” (the one that was found to have the biggest spatial influence, as shown in the analysis section). Considering all neighbourhood types of all longitudinal trajectories, we consider two different scenarios. In Scenario 1, we randomly select five neighbourhoods if they are classified as “towers” in the fourth year, i.e., *u*_*t*=4_, and change them to “mixed suburban” in year five, *u*_*t*=5_ = “mixed suburban”, for all selected neighbourhoods. We select a relatively small number of intervention cases because this action is not trivial in practice, plus it helps to evaluate the impact of small thresholds of change. In Scenario 2, we randomly select one tract classified as “towers” in year four, and the immediate four nearest neighbours of the same type also in year 4, and change all of them to “mixed suburban” in year five. While the non-spatial scenarios allow us to examine threshold effects, these spatial scenarios investigate geographical distributional effects. Moreover, these scenarios allow us to examine how small changes in the initial distribution of neighbourhood types affect the long term evolution of the city, even if its transition probabilities are not altered.

With these adjustments in the longitudinal trajectories for both scenarios, we create a new spatial Markov chain $S^{u^{\prime }}$, following the same steps for the spatial (non-counterfactual) case. We also perform predictions about the distribution of neighbourhood types 50 time steps in the future, obtaining $P^{u^{\prime }}$. To do so, we use *π*(*t* = 5) to make predictions based on $S^{u^{\prime }}$. This whole process is repeated 100 times, and we work with their average values: $\frac{1}{100}\sum_{i=1}^{100}P_{i}^{u^{\prime }}$ for every neighbourhood type *u* ∈ *U*.

## Results

Our analysis proceeds according to the three research questions articulated above.

### What are the main types of neighbourhoods in the city and how are they organized, spatially and hierarchically?

A challenge in any clustering task is the number of groups (or clusters) to feature, and there is no simple solution. Many neighbourhood clustering studies use various statistical techniques as guides, such as within-cluster sum of squares or the silhouette method. In neighbourhood research, these tend to suggest a relatively small number of clusters, often between 4 and 7. For this study, we chose to highlight a somewhat larger number, beginning our search in the 10-15 range. This allows us to retain the benefit of multidimensional clustering—the ability to identify latent meanings arising from combinations of factors—while remaining closer to the underlying variables. With a small number of clusters, changes in these variables may not appear.

**Fig 2:**
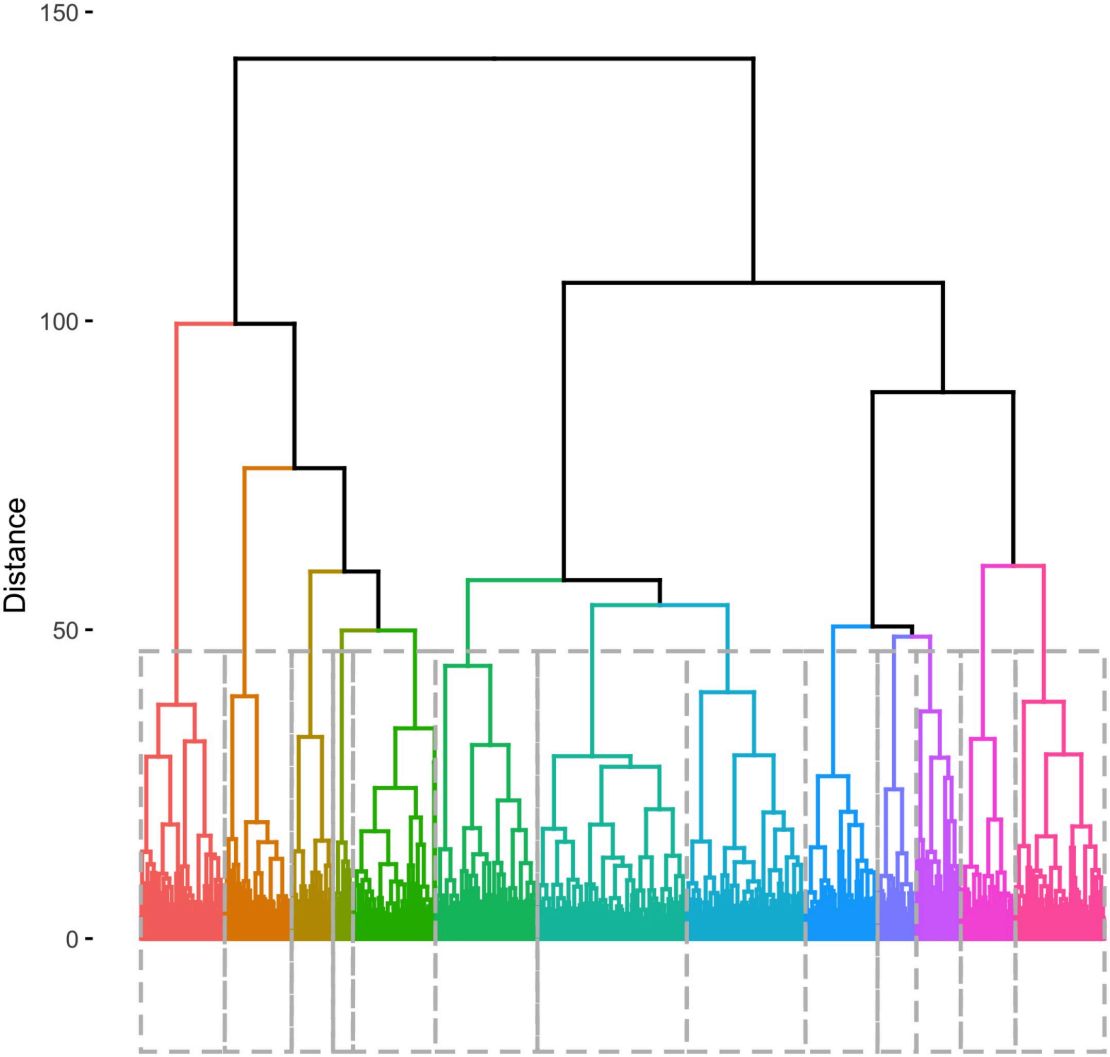
Dendrogram of clustering results. This figure shows the dendrogram generated by the hierarchical k-means clustering process described in the methodology section. The regions of the figure marked by dashed rectangles and colours show the thirteen clusters selected for deeper analysis in this study. In other words, each coloured area of the dendrogram includes the set of census tracts associated with the thirteen neighbourhood types listed in Table 2.

To select the specific number of clusters for our analysis, we examine the dendrogram represented in Fig 2.

Looking at the clusters in the 10-15 range (height around 50), the dendrogram suggests focusing on 13 or 14 clusters. We qualitatively examined both of these solutions, and found that the 14 cluster solution did not add significant analytical value in the present context and so opted for the more parsimonious 13 cluster solution. In the 14 cluster solution, the predominantly Asian portions of the city’s inner suburbs, which are largely geographically contiguous, are further subdivided, mostly by socioeconomic status. Examining neighbourhoods at this level of specificity could be valuable for further research focused on this segment of the city. At the same time, we situate the 13 clusters within the context of the city’s higher-order structure, three larger groups, as suggested by the dendrogram. Particularly, for this interpretation task, we use hierarchical clustering and also t-SNE, confirming the distinct separability of three groups (see S1 and S2 Figs).

Table 2 summarizes the neighbourhood typology that emerges from the clustering process. Radar plots with complete results are in the (S3 Fig).

**Table 2:** Hierarchical typology of Toronto neighbourhoods.

**Creative City**	
**High**	**Low**
arts & culture, science and tech, education, density, high rises, youth, renters, movers.	drivers, detached housing, unemployment, married couples, black, south asian, service workers, blue collar
**Established Creative, Mixed Creative, Young Urban Professionals, Portuguese Predominant**	
**Suburban**	
**High**	**Low**
managers, business administration, science and tech, drivers, income, education, home value, detached housing, seniors, married couples	service workers, blue collar, government income, density, high rises, youth, renters, movers, black, south asian, unemployment
**Elite Suburban, Mixed Suburban, Chinese Predominant, Jewish Predominant, Greek Predominant**	
**Marginalized**	
**High**	**Low**
service workers, blue collar, government income, renters, black, south asian, unemployment, high rises	managers, arts & culture, business admin, science & tech, income, education, home value, detached housing
**Towers, Black Predominant, South Asian Predominant, Italian Predominant**	

**Fig 3:**
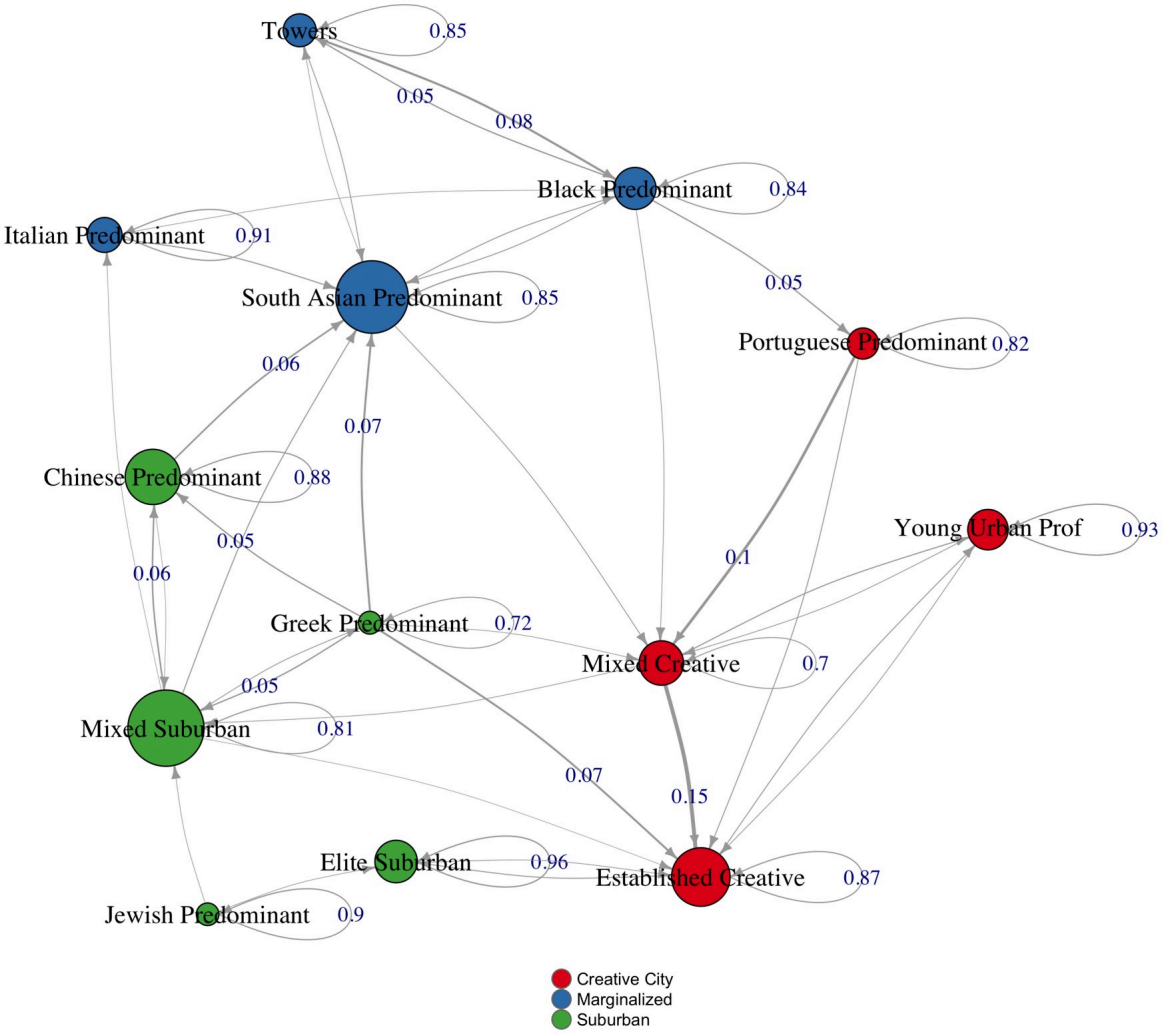
Network representation of neighbourhood transition probabilities. This figure summarizes the transition probabilities across neighbourhood types as a simplified network. To increase legibility, the figure excludes transitions with a probability less than or equal to .02 and only labels probabilities greater than or equal to .05 on edges. Edge widths are proportional to transition probabilities (excluding self-loops), and nodes are coloured to correspond to the higher-order structures of which each neighbourhood is a member. Node sizes are proportional to the number of census tracts of that type.

This table summarizes the multilevel neighbourhood typology that emerges from our cluster analysis. The typology divides the city into three higher order types of neighbourhoods, “Creative City,” “Suburban,” and “Marginalized,” which are characterized primarily by high and low values on the variables listed below each label. As Fig 3 shows, these types also exhibit a higher degree of similarity. More specific neighbourhood types that fall within each of these higher-order classifications are listed below them (in bold).

Overall, Toronto is characterized by three main types of neighbourhoods with various subtypes:

1. “Creative city” neighbourhoods tend to be located in the downtown core and resemble the types of neighbourhoods featured in Richard Florida’s “The Rise of the Creative Class” [68]: high density areas populated by mobile, highly educated young people, singles, artists, science and tech workers, with relatively few low income blue collar and service workers. More specific types include “established creative” neighbourhoods with very high concentrations of art & culture workers, high home values, single family homes, few blue collar and service workers, and relatively low density; “mixed creative” areas, located at the fringes of the downtown core, that exhibit more occupational and ethno-racial diversity; and “young urban professional” neighbourhoods featuring very high concentrations of highly educated young professional singles focalized in the city center. Toronto’s predominantly Portuguese neighbourhoods continue to have a foothold in this part of the city.
2. “Suburban” neighbourhoods, by contrast, are high income, low density areas with fewer youth and more homeowners, high home values, much detached housing, and numerous managers and businesspeople. This part of the city is largely made up of neighbourhoods defined by socioeconomic status and ethnicity. “Elite” suburban neighbourhoods have the city’s highest incomes, most managers, lowest density, highest home values, and fewest blue collar and service workers, while “mixed” suburban areas have middle incomes and greater occupational and ethnic diversity. Other suburban neighbourhoods are defined more by a predominant ethnic community: Greek, Jewish, or Chinese.
3. The city’s “marginalized” areas, also located generally outside the downtown core, tend to be characterized by lower income non-white residents, higher unemployment, more blue collar and service workers, lower home values, and high-rise towers. This part of the city contains dense, high-rise social housing complexes –“towers”– marked by high unemployment, high turnover, and concentrations of diverse non-white immigrant communities (especially Arab, South Asian, and Black residents). Other working class neighbourhoods in this part of the city are also ethnically diverse but also have a relatively predominant ethnic group: Black, South Asian, or Italian.

This typology resembles other neighbourhood classifications of Toronto derived by different methods [3, 58]. These also identify three major clusters of neighbourhoods with similar geographical, socioeconomic, and demographic patterns. Our typology adds specificity by identifying subtypes within these major patterns and allowing us to examine the complex multilevel interactions between the higher-order structure of the city and its lower-level constituents.

### What are the main patterns of neighbourhood change over time, and how are they inflected by space?

Having identified the structure and semantic of the city’s neighbourhoods, we turn to our second research question. To answer this question, predictions are made based on the Markov chain generated from the data describing transition probabilities among the 13 neighbourhood types listed in Table 2, as described in the methodology. To validate the performance of the Markov model, we first used it to predict the distribution of these thirteen types at the last (fifth) time point for which we have data. This fifth time period was hidden on purpose in the Markov chain construction so we could access the quality of our predictions. As Table 3 shows, we find very low errors in all the experiments, with consistently lower RMSE values than the baseline models (the only exception is predictions based on the identity matrix from year 4). These results indicate that, despite the relatively few time points, our model is robust to predict the distribution of neighbourhood types in the near-term future. After these validation steps, we used the Markov chain generated from all five time points (years 1-5) to generate predictions 50 time steps in the future to examine how the city would evolve if these trends continue and as part of our counterfactual scenarios.

**Table 3:** Evaluating the error of the model.

Predictor	Real	Identity	Random
*t* = 1	0.00848	0.02313	0.11657
*t* = 2	0.01016	0.01519	0.11573
*t* = 3	0.01063	0.01261	0.11556
*t* = 4	0.00762	0.00571	0.11569

Values in the table represent a RMSE from all predictions exploring different predictors: *t* = 1 is the state on year one, i.e., *π*(*t* = 1); *t* = 2 is the state on year two, *π*(*t* = 2); *t* = 3 is the state on year three, *π*(*t* = 3); and *t* = 4 is the state on year four, *π*(*t* = 4). For the random experiment, all confidence interval values for each predicted neighbourhood type were below 0.001, i.e., the predictions obtained do not change significantly, and they were omitted to favor legibility. Real refers to the results with real data, Identify to the results with an identity matrix, and Random to the results for the random experiment.

Fig 3 depicts the transition matrix as a network (the full matrix is in the S4 Fig). As in previous neighbourhood change studies, the dominant pattern is continuity. Most of the time, neighbourhoods remain similar from one year to the next: all have at least a .7 probability of reproducing themselves, and most (9/13) are above .85. In this way, each new iteration of the city is at once the same and subtly different from its predecessor. Dramatic change is rare, but for that reason it stands out all the more when it occurs. This helps to explain why the predictions based on the identity matrix considering year 4 to predict year 5 have a good performance.

The most stable types of neighbourhoods are “elite suburban” and “young urban professional,” while the least stable are mixed (suburban and creative) or European ethnic neighbourhoods (Greek, Portuguese). These latter tend to transition into either “mixed” or “established creative” neighbourhoods, while “mixed creative” neighbourhoods, in turn, are relatively unlikely to sustain themselves. Instead, they are highly likely (.15) to transition into “established creative” types. If we think of this pathway as gentrification, then the most likely targets are older European immigrant neighbourhoods. Nevertheless, in virtue of their somewhat interstitial position (they are located in the Western and Eastern transitional zones between the “creative city,” “suburban,” and “marginalized areas”) these neighbourhoods are subject to a diversity of transitions. For example, Greek predominant neighbourhoods tend to transition into “South Asian predominant,” “Chinese predominant,” and “established creative” areas—spanning all three higher-order types. By contrast, “mixed suburban” neighbourhoods, which also show a high degree of volatility, tend to transition into newer immigrant areas, which show little propensity to gentrify. This overall pattern whereby middle class suburban areas are diminishing while high status suburban and downtown youth areas persist matches other recent studies of Toronto [69]. At the same time, the prevalence of horizontal transitions—“Chinese predominant” with “mixed suburban,” “towers” with “black predominant” illustrate the diversity of forms of change identified in past US studies.

While the city exhibits a high degree of continuity, its degree of structure varies. Rather than assume there is an equally powerful structure at work throughout, an important question concerns how much of a given urban environment is structured at all, and to what degree. We find that much of Toronto is very deeply structured, so that it is highly likely to reproduce itself. In fact, some of the most “creative” parts of the city in terms of who is there and what they are doing—areas in which young, highly educated arts and technology workers predominant—are the most stable. By contrast, other parts of the city exhibit creativity where the urban fabric itself is in a state of transition in which neighbourhood forms themselves rise and fall more rapidly. There are, therefore, at least two types of urban creativity at work here. One appears to thrive within a stable urban context that supports a specific set of groups and activities; in the other, the urban form itself is in a more fluid state of experiment and transformation.

**Fig 4:**
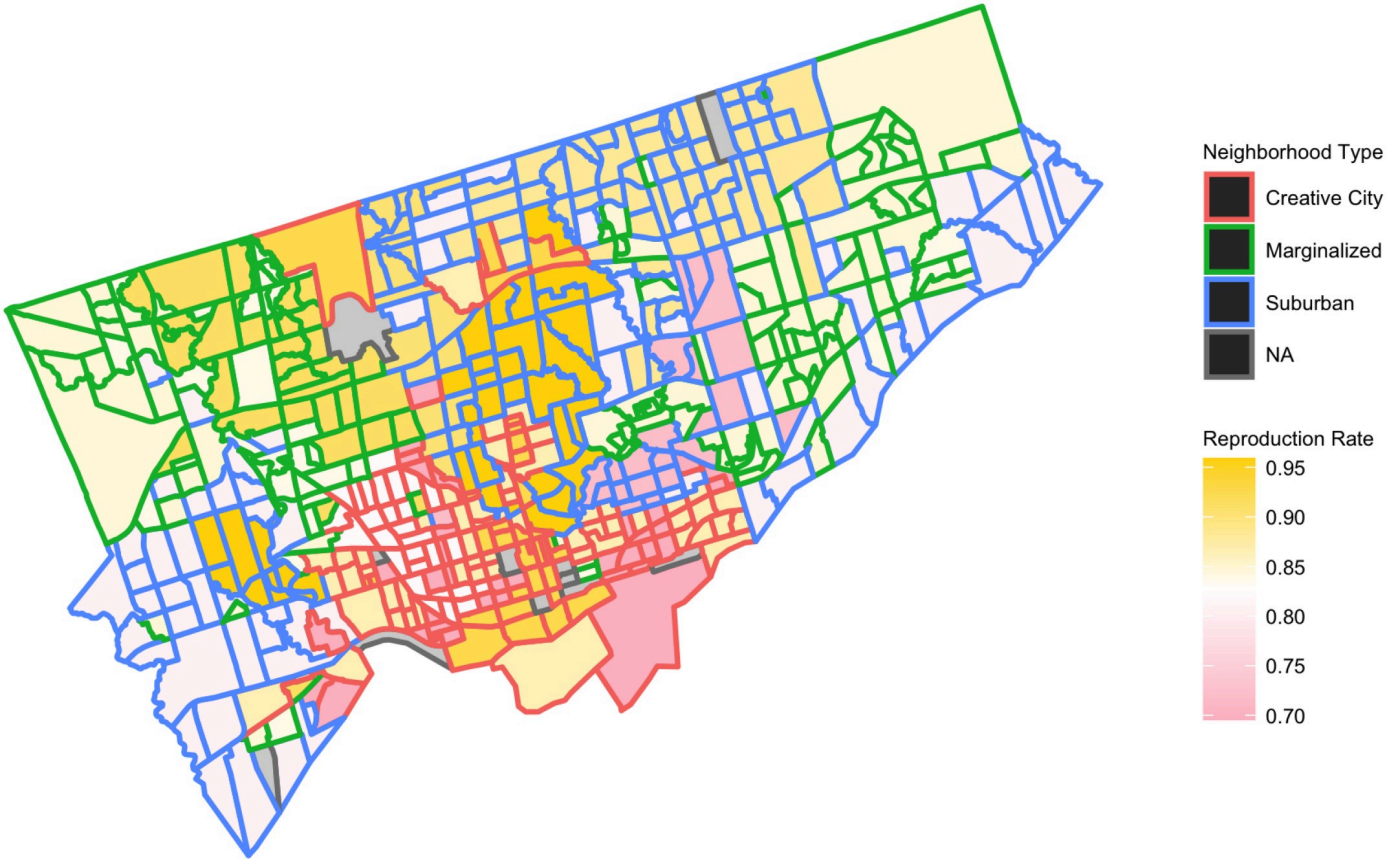
Map of neighbourhood reproduction rates. This figure maps the reproduction rates of neighbourhoods across the city“Reproduction rates” refer to the probability that a given neighbourhood type will recur, that is, the diagonal value in the transition matrix. In this map, each census tract is assigned its neighbourhood type’s diagonal value, according to the thirteen neighbourhood types listed in Table 2. Pink areas are more volatile, and yellow areas are more stable. Tract borders are coloured to indicate each tract’s position in the higher-order typology depicted in Table 2.

These results reveal a complex hierarchical structure governing urban evolution. The major ordering principles of the city reproduce themselves and maintain the differences between them, even as there is a relatively large amount of movement to and from different local formations within them. The transition probabilities for the higher order principles (creative city, suburbs, marginality) are all above .9. Moreover, as Fig 4 shows, the greatest stability through time tends to be located at the geographic center of these areas. At the same time, amidst these “high fidelity” zones, there are smaller zones of volatility. They are generally located in interstitial areas near the edges of the three higher-order zones, that is, where “creative city” borders “marginalized” and “suburban” areas or “marginalized and “suburban” border one another. This spatio-temporal pattern reveals an important dimension of urban complexity that has been highlighted in computer simulation studies of the self-organization of cities [48]: zones of local instability within a more global spatio-temporal stability, where the areas “in between—the boundaries—are thus the most critical areas in the city for socio-spatial changes (and in society at large).” This description of an artificially generated city is remarkably similar to the one we have drawn from real-world data on Toronto.

To investigate these spatio-temporal dependencies more precisely, we generate a spatial Markov chain according to the methodology described above. Full results are shown in the Supporting Information. Before we proceed, we evaluate the influence of neighbourhood type on transition probabilities on immediate spatial surroundings (our spatial case). For that, we use a test of spatial independence, as described in the methodology section. Our results show that spatial dependence in terms of surrounding neighbourhood type has a statistically significant impact on transition probabilities. According to the *χ*^2^ test, the system is not independent across space because the test statistic is *Q* = 2354.1 (*p* < 0.001, *DoF* = 854), thus, rejecting the null hypothesis of spatial independence. This means that a state’s transition cannot be assumed to be independent of the neighbourhood type of its neighbours.

**Fig 5:**
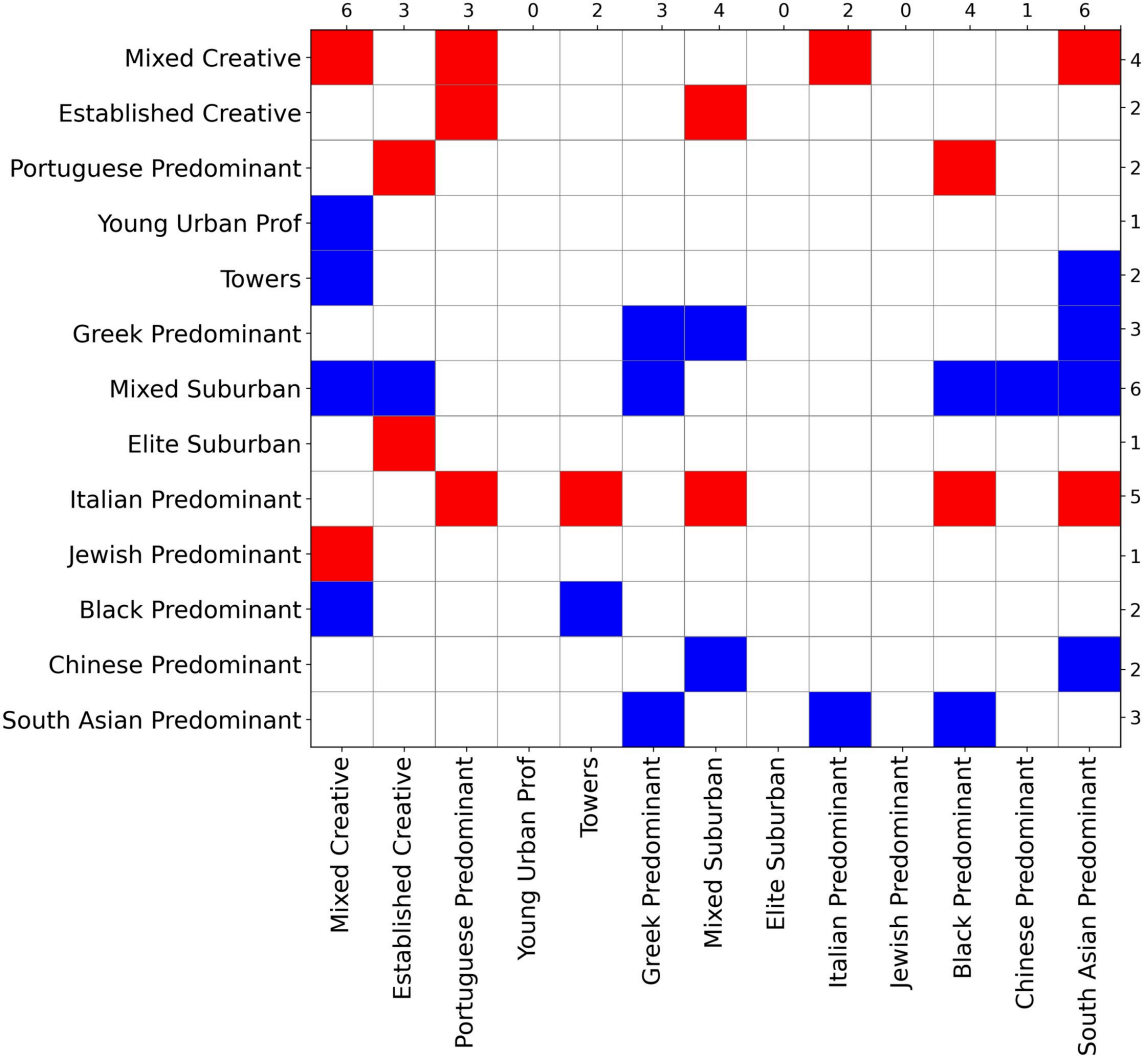
Spatial markov model results regarding differences on reproduction rates. This figure represents an increase (blue) or decrease (red) of at least 5% on the reproduction rate (diagonal value) on the spatial case compared to the non-spatial case. Numbers on top and right are the total counts of colored cells for each column and line, respectively.

Next, we highlight some overall patterns and particularly interesting findings in Fig 5. Given the high degree of spatial clustering, many neighbourhood types are never or rarely nearby, and therefore the number of possible spatially dependent transitions is often low. We, therefore, restrict the analysis to transitions that occur at least ten times, causing an increase or decrease of at least 5% on the reproduction rate (diagonal value) compared to the non-spatial case.

Five key results are depicted in Fig 5:

- First, spatial dependence is high in general. Across most neighbourhood types, the reproduction rate shifts substantially depending on its neighbours. This was the case for 10/13 neighbourhood types, as we can observe by looking at the columns of Fig 5. For instance, the neighbourhood type “mixed creative” is influenced in this regard by six different types of neighbours.
- Second, some neighbourhood types show more spatial dependence than others. Types “south asian predominant” and “mixed creative” show the greatest tendency to become more volatile in the presence of neighbours, while “young urban professionals”, “elite suburban”, and “jewish predominant” neighbourhood types are less sensitive to their surrounding geographic context (their reproduction rates do not significantly change depending on nearby neighbourhood types).
- Third, some neighbourhood types induce greater increases or decreases in the reproduction rates of their neighbours. “Jewish predominant,” “Italian predominant,” “elite suburban,” “Portuguese predominant,” “established creative,” and “mixed creative” induce significant decreases in reproduction rates of their neighbours (red colours in Fig 5); by contrast, the remaining neighbourhood types favour increasing stability in their surroundings (blue colour in Fig 5), for instance, “towers” enhances the stability of “mixed creative” and “south asian predominant.”
- Fourth, besides diagonals, some particular transitions are especially affected by their spatial location. In particular, the transition probability “Portuguese predominant” → “mixed creative” shifts from 0.103 to 0.188 when near “mixed creative” and the transition probability “mixed creative” → “established creative” shifts from 0.146 to 0.205 when near “established creative.” These are situations where neighbourhood change exhibits spatial tipping, in which the appearance of a neighbouring type harkens impending nearby transformations, in particular in these cases toward the spread of a specific type (“mixed creative” or “established creative”) to its neighbours.
- Finally, the city’s multilevel neighbourhood order affects the spatial dependencies of its temporal evolution, though this impact varies. In general, “marginalized” neighbourhood types become more volatile when they are near “creative city” neighbourhoods, but the reverse happens less frequently. This adds more specificity to the results depicted above in Fig 4.

Altogether, these findings provide strong support for the view of urban evolution advanced by complexity theories of cities. Simultaneous stability and openness arise from a confluence of top-down and bottom-up processes. Zones of local volatility are globally stable, and patterns of global change contain local stability. The temporality of urban evolution is grounded in space, just as the spatial arrangement of the city emerges from its neighbourhoods’ divergent trajectories through time.

### How are these trends likely to unfold in the future, and how might they change under certain urban planning scenarios?

**Fig 6:**
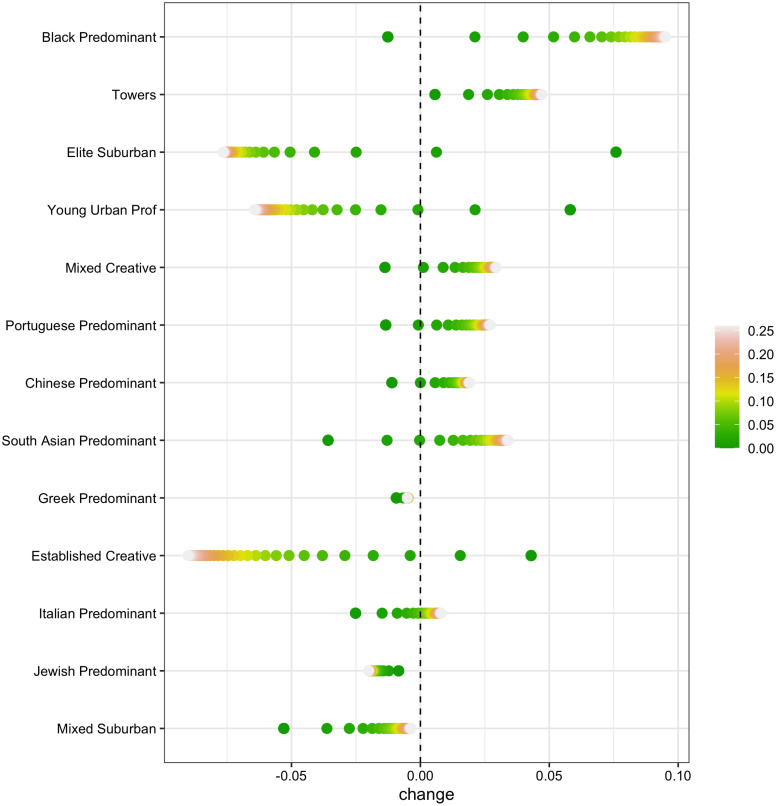
Predicted changes in neighbourhood distribution according to multiple scenarios. This figure summarizes predicted changes in the overall distribution of neighbourhood types in 50 time steps for multiple models, compared to the distribution at t5. 0 represents no change to current transition probabilities (darkest green dots) and shows the non-counterfactual scenario in which the city continues to evolve according to its current pattern. Dots positioned further to the right or left in Fig 6 indicate divergence from the distribution at t5, which is represented by the vertical dashed line. Lighter shaded dots represent increasingly larger changes to transitional probabilities, as outlined in the methodology section. As a robustness check, we explored fewer and more time steps, and the same pattern was observed, just less/more extreme.

To address our third and final research question, we use the Markov chains described above to predict how the city is likely to evolve in the future, and how this evolution would change under different scenarios. Fig 6 shows the difference between the predicted distribution of neighbourhood types and the actual 2016 distribution in 50 time steps, along with two counterfactual scenarios described in the methodology section.

The non-counterfactual results (0 change, the darkest green dot) in Fig 6 show how the city would evolve if it continued forward according to its current trajectory. The dominant trend would be increased socioeconomic polarization: for example, elite suburban neighbourhoods would increase their share of the overall population of neighbourhoods by around 7.5 percentage points (from 8 to 15.5%), and “young urban professional” and “established creative” areas by around 5 percentage points (from 11 and 7 percent, respectively). All together, these three upper status areas would grow from around 26 to 35% of the city as a whole. At the same time, middle income diverse suburban neighbourhoods would decline (by about 5 percentage points), along with most of the city’s ethnic working and service class communities, as well as its “mixed creative” neighbourhoods. If unchecked, current trends point toward a solidification of the “divided city” [70].

A benefit of our methodological approach is that it allows us to envision alternative scenarios that might change this trajectory towards polarization and division. Three key points stand out in examining the counterfactual scenarios in Fig 6. First, in line with complexity theories, small initial changes can have big effects. In both scenarios, the growth of “young urban professional,” “elite suburban,” and “established creative” areas is substantially reduced. By contrast, the decline in the city’s occupationally and ethnically diverse areas is reduced or stabilized in “mixed creative,” “chinese predominant,” “portuguese predominant,” and “south asian predominant” areas. In some cases, such as predominantly black neighbourhoods, the trend reverses to net growth. Second, we see some signs of non-linear thresholds, again in line with complexity theories of cities. The incremental change from a .01 change in transition probabilities to a .02 change in transition probabilities generates relatively sharp downstream effects, most strikingly in the case of “young urban professional,” “elite suburban,” and “black predominant” neighbourhood types. However, the effects are non-linear and diminish at higher levels. For example, there is very little difference in the effect of a change from 12% vs. 13%. This non-linearity makes sense in the context of these specific scenarios: we are altering transitions that in the non-counterfactual scenario are very rare. Therefore, lower values (e.g. 1% or 2%) represent the initial introduction of a process that rarely occurred previously. As values increase, the process is in place, and additions do not change the situation as much beyond a certain threshold. The bunching in Fig 6 at higher values shows us approximately where this threshold is for the scenarios in this experiment. And third, we see evidence of indirect effects characteristic of complex systems. While we did not make any change to the transition probabilities for “south asian predominant” or “tower” neighbourhoods, their relative footprint in the city grew compared to the non-counterfactual scenario.

All in all, these results show that in a complex dynamic interacting system, small quantitative changes at critical points can potentially make a substantial qualitative difference. Connecting disconnected and divided upper status areas with lower status areas reduces the isolation of these parts of the city, and helps others to retain their foothold. This, in turn, reveals another sign of a complex system: changes in one part reverberate in others.

**Fig 7:**
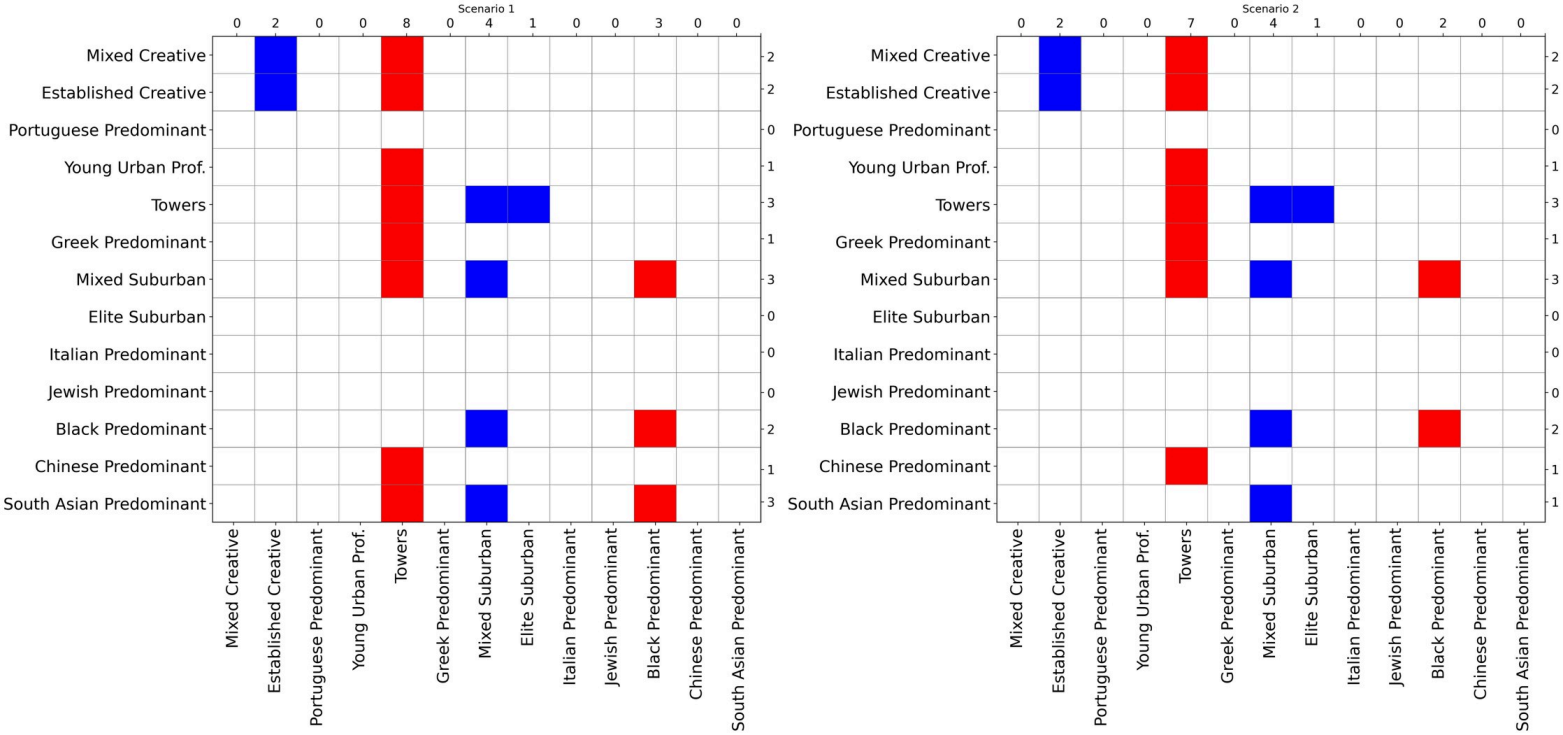
Results of counterfactual scenarios for the spatial case. This figure summarizes the spatial counterfactual scenarios’ results (Scenarios 1 and 2), showing where there is a difference of at least 1% in relation to the non-counterfactual case when predicting the distribution of neighbourhood types: blue (at least 1% increase) and red (at least 1% decrease). Labels on the y-axis left indicate the conditioned neighbourhood type on the spatial Markov over all other ones (x-axis bottom). Numbers on top and right are the total counts of coloured cells for each column and line, respectively. As a robustness check, we explored fewer and more time steps, and the same pattern was observed, just less/more extreme.

Fig 7 summarizes the results of the spatial counterfactual scenarios described in the methodology. Here we consider the effects of changing five tracts from “towers” to “mixed suburban,” while leaving other transition probabilities unchanged. In Scenario 1, the five tracts are randomly distributed around the city; in Scenario 2, they are geographically clustered. These situations allow us to examine the impacts of the initial distribution of neighbourhood types on the evolution of their geographic arrangement. For both scenarios, the figure shows where there is a difference of at least 1% in relation to the non-counterfactual case when predicting the distribution of neighbourhood types. More specifically, if in the non-counterfactual case the model predicts a given neighbourhood type will constitute 10% of the total tracts nearby the target type, and in the counterfactual case the distribution is greater than 11% or less than 9%, we colour the appropriate box in the matrix: blue (at least 1% increase) and red (at least 1% decrease). Rows indicate the conditioned neighbourhood type on the spatial Markov over all other ones (columns).

Fig 7 indicates a substantial similarity between the two scenarios, as we would expect. The median value regarding the distribution of all differences between the same transition probabilities in Scenario 1 and Scenario 2 is 0, with a standard deviation of 0.0066. Also, there is no difference bigger than 3% compared to the non-counterfactual case in any scenario. Nevertheless, some nuances emerge on closer inspection. For instance, while the growth of “mixed suburban” in both scenarios is highly similar, Scenario 1 (Fig 7-left) induces declining shares of “towers” and “black predominant” neighbourhoods in the geographic context of “south asian predominant,” with all the averages statistically different under a 95% confidence interval. In addition, the reproduction rate of “black predominant” is higher on Scenario 1 (average of 0.192 with + −0.008 95% CI) compared to Scenario 2 (average of 0.206 with + −0.002 95% CI). In both scenarios, we note a slight tendency toward a potential form of gentrification. For example, we observe a considerable increase in the probability of a neighbourhood of type “established creative” having a neighbour of type “mixed creative.”

These results suggest that introducing a small change in the initial spatial distribution of neighbourhood types can lead to overall urban transformation, and this tends to be enhanced if these changes are more widely spread than concentrated in one geographic area.

## Discussion and conclusion

Our paper has sought to advance the literatures on socio-spatial neighbourhood change and cities as complex systems in different aspects. First, we applied neighbourhood change classification techniques to an important case, Toronto, where it has not been fully explored previously. Second, we show the value of incorporating a multilevel neighbourhood classification approach. Not only does this approach permit the analyst to incorporate more neighbourhood types than is typical in the literature, but it also allows the observation of distinct patterns of temporal change, where changes are focalized at interstitial zones in the typology. Third, we show how to exploit formal properties of Markov models for neighbourhood change research. Whereas prior research has used Markov models to identify transition patterns, we advance the literature by illustrating how to 1) validate their predictive power and 2) utilize them to explore counterfactual thought experiments for evaluating the potential consequences of various planning interventions. Fourth, we advance the literature on cities as complex systems by elaborating techniques for empirically applying to urban neighbourhood data key complexity concepts—in particular non-linearity and threshold effects—and showing how to develop scenario based thinking with such data.

While this study has made a number of significant contributions, it is not without limitations. These point toward important areas of future research. One limitation concerns temporal and geographic scope. Carrying these methods further into the past and into comparative studies of other cities and countries would greatly enhance their value. Doing so is challenging because of data comparability issues. Recent advances have helped to overcome the challenge of changing definitions of neighbourhood borders [35] but changing variable definitions (i.e. in occupational or ethnic labels) remain a major obstacle. Solving this problem would enable longer-term studies at a fine-grained level. Another limitation concerns the use of first-order Markov chains. These by definition do not consider memory of past transitions, which in the urban context is likely considerable. Future research, especially of longer-term trajectories, would benefit from incorporating higher-order Markov chains. Our models moreover only enable the prediction of the distribution of neighbourhood types in a particular city. For applications requiring more granularity, a different methodology would be required. Despite obtaining strong evidence that there is spatial dependence in the neighbourhood socioeconomic evolution, which could be helpful in many ways, this study would also benefit from further research trying to empirically explain and prove causation regarding those patterns.

Though the primary ambition of this study is not theoretical, we lay the ground for more fulsome efforts at theoretical synthesis. For example, while we have pursued a Markovian approach oriented toward generative processes rather than overall sequences, synthesizing these two approaches rather than placing them in competition with one another strikes us as an important way to move the field forward, both theoretically and empirically. A related and exciting direction for future research is to bring recent innovations from general social sequence analysis into the domain of neighbourhood change research. Cornwell [37] suggests that joining network methods and sequence analysis offers one of the most promising “new directions” in this regard, especially through exploiting properties of two-mode networks (i.e., affiliation networks). In the context of neighbourhood change research, a first step towards this synthesis could be to treat the neighbourhood types produced through geodemographic segmentation as one set of nodes and actual neighbourhoods as another set in a two mode-network. In this way, in one mode, neighbourhood types are connected when actual neighbourhoods transition between them. In the other mode, concrete neighbourhoods are connected in virtue of undergoing the same transition between types. Exploring the properties of the resulting networks (such as their density, centrality, and so on) could reveal aspects of neighbourhood change missed by standard methods. Further work in this direction could examine evolving neighbourhood networks generated by activity patterns of individuals [71] to classify neighbourhoods. Emerging work uses, for example, social media data to identify static snapshots of neighbourhoods [72, 73], but examining dynamic processes may be pursued through other data sources, such as government administrative records [74]. Pursuing these nascent lines of research offers an important avenue for carrying forward the work of this study.

## Supporting information

S1 Fig Hierarchical clustering on clusters’ centroids representing neighbourhood types. This figure shows a dendrogram representing the arrangement of neighbourhood types in hierarchical clustering (using Ward linkage criteria and cosine distance). The dashed line represents a cut indicating the presence of three distinct clusters.
(TIF)

S2 Fig Visualization of high-dimensional clusters’ centroids representing neighbourhood types in 2-dimensions. This figure represents the result of t-SNE on the clusters’ centroids of neighbourhood types. It is possible to identify three distinct groups (information robust with different t-SNE configurations).
(TIF)

S3 Fig Radar plots of clusters. This figure shows a radar plot of how the 28 census variables included in the clustering algorithm (shown in Table 1) relate to the thirteen clusters examined in this study (summarized in the methodology section). Points in the radar plot indicate average levels of a given variable for each cluster’s center. Because all variables are standardized, the average is 0, which is indicated by the dashed blue circle. For example, the “Established Creative” cluster contains about an average level of Portuguese residents (“eth.port.s”). Points toward the outer rim of the plot show levels considerably higher than the city average, with the number at the outer rim showing the upper limit for that plot (in this case, a z-score of 4). Points closer to the center are lower than the city average. The descriptions of the specific neighbourhood types in the text (e.g. “Elite Suburban” or “Portuguese Predominant”) are based on these plots. For example, they characterize neighbourhood types that stand out for one group (such as the high level of Portuguese residents in “Portuguese Predominant”) or a mix (such as “Elite Suburbs,” which have the city’s highest incomes, most managers, lowest density, highest home values, and fewest blue collar and service workers).
(TIF)

S4 Fig Non-spatial and spatial Markov chains. Heat maps representing the non-spatial Markov chain and all the thirteen spatial Markov chains, according to the names indicated on each figure. For instance, the figure with the title “Spatial—Neighboring Mixed Creative” represents the spatial Markov chain whose transitions are conditioned to the neighbourhood type “Mixed Creative”.
(TIF)
