# Supplementary figures and images for: A Markov model of urban evolution: Neighbourhood change as a complex process
Source: PLoS One. 2021 Jan 15;16(1):e0245357. doi: 10.1371/journal.pone.0245357 (PMC7810345; doi:10.1371/journal.pone.0245357)

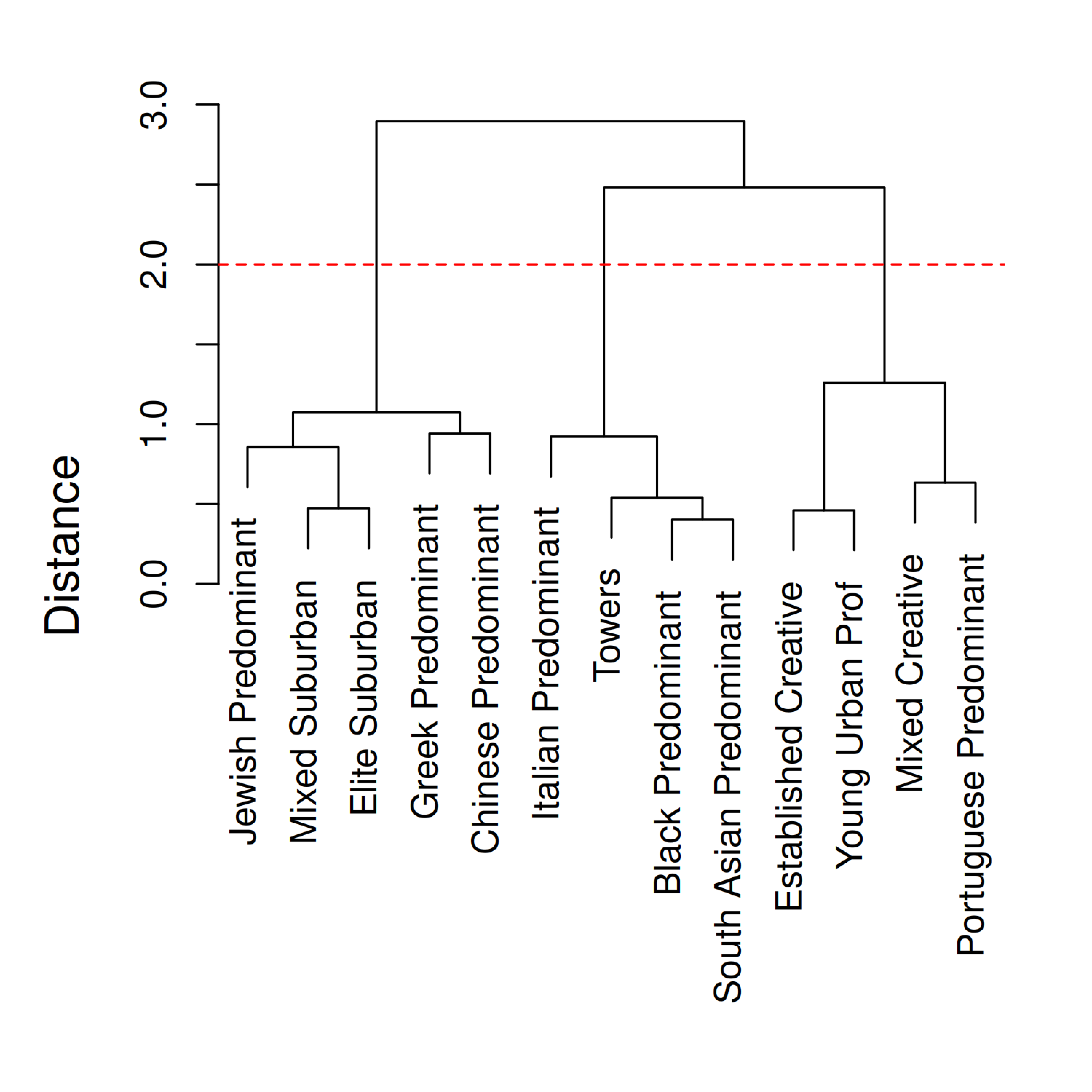

Supplement: S1 Fig — This figure shows a dendrogram representing the arrangement of neighbourhood types in hierarchical clustering (using Ward linkage criteria and cosine distance). The dashed line represents a cut indicating the presence of three distinct clusters. (TIF) [file pone.0245357.s001.tif]

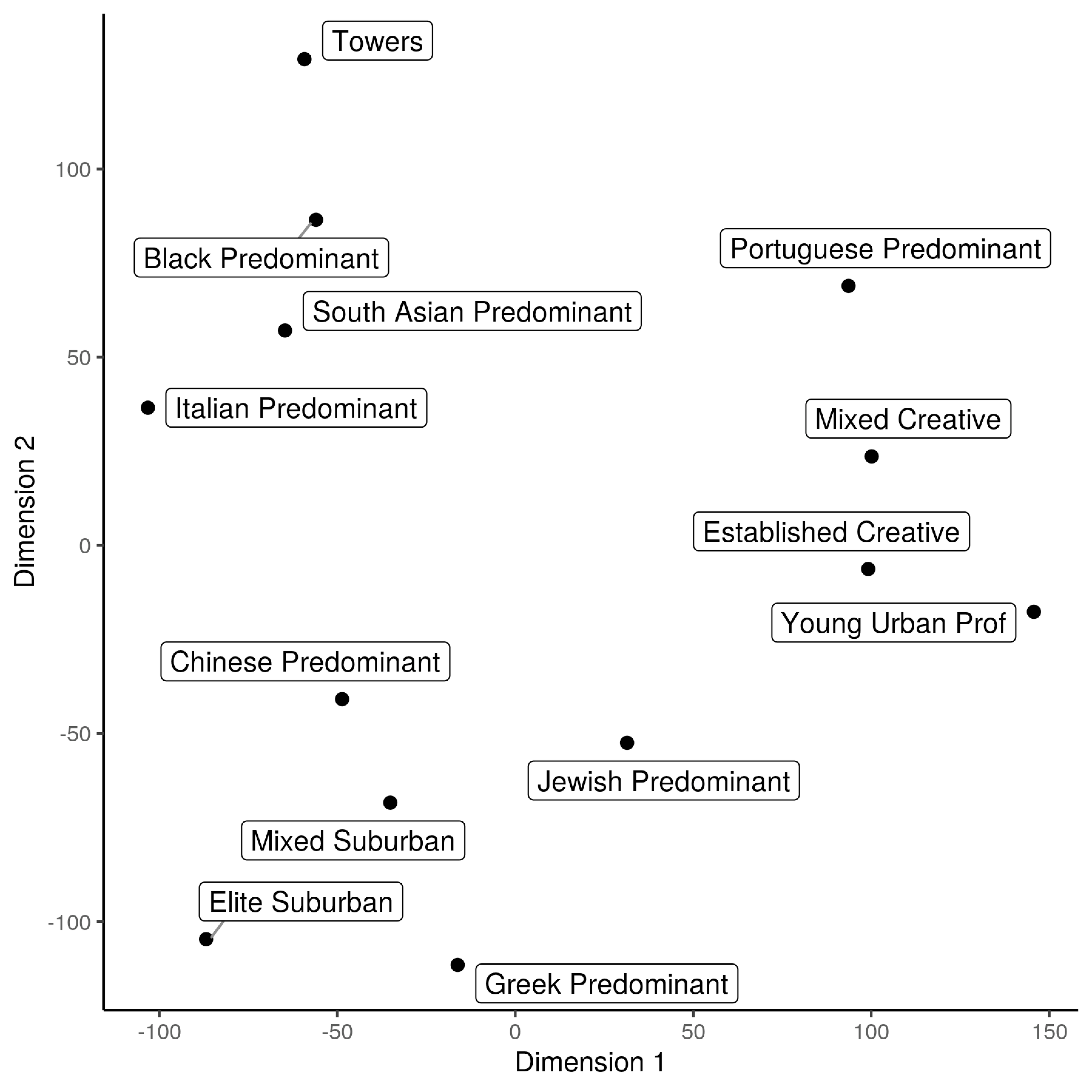

Supplement: S2 Fig — This figure represents the result of t-SNE on the clusters’ centroids of neighbourhood types. It is possible to identify three distinct groups (information robust with different t-SNE configurations). (TIF) [file pone.0245357.s002.tif]

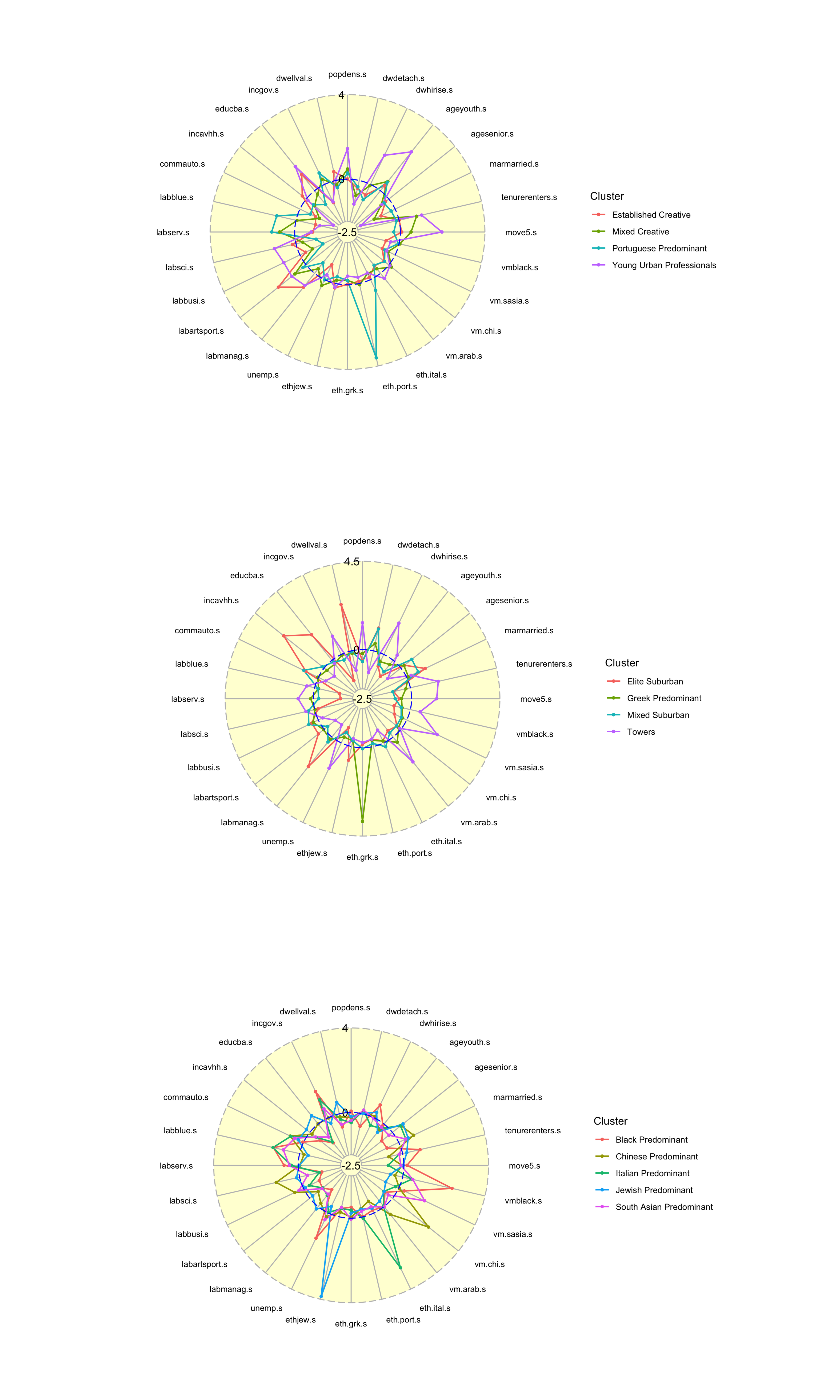

Supplement: S3 Fig — This figure shows a radar plot of how the 28 census variables included in the clustering algorithm (shown in Table 1) relate to the thirteen clusters examined in this study (summarized in the methodology section). Points in the radar plot indicate average levels of a given variable for each cluster’s center. Because all variables are standardized, the average is 0, which is indicated by the dashed blue circle. For example, the “Established Creative” cluster contains about an average level of Portuguese residents (“eth.port.s”). Points toward the outer rim of the plot show levels considerably higher than the city average, with the number at the outer rim showing the upper limit for that plot (in this case, a z-score of 4). Points closer to the center are lower than the city average. The descriptions of the specific neighbourhood types in the text (e.g. “Elite Suburban” or “Portuguese Predominant”) are based on these plots. For example, they characterize neighbourhood types that stand out for one group (such as the high level of Portuguese residents in “Portuguese Predominant”) or a mix (such as “Elite Suburbs,” which have the city’s highest incomes, most managers, lowest density, highest home values, and fewest blue collar and service workers). (TIF) [file pone.0245357.s003.tif]

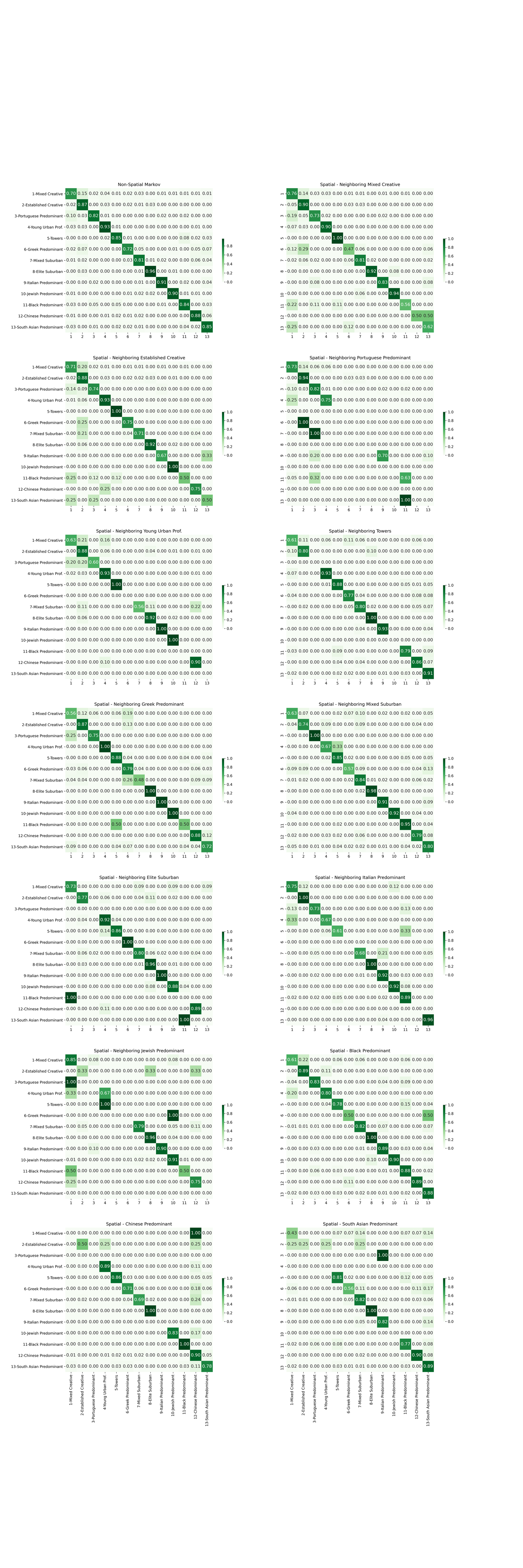

Supplement: S4 Fig — Heat maps representing the non-spatial Markov chain and all the thirteen spatial Markov chains, according to the names indicated on each figure. For instance, the figure with the title “Spatial—Neighboring Mixed Creative” represents the spatial Markov chain whose transitions are conditioned to the neighbourhood type “Mixed Creative”. (TIF) [file pone.0245357.s004.tif]
